# A thymine-challenge test to prospectively evaluate dihydropyrimidine dehydrogenase activity for risk of severe 5-fluorouracil-induced gastrointestinal toxicity

**DOI:** 10.1007/s00280-025-04804-6

**Published:** 2025-08-18

**Authors:** Nuala Helsby, Katrina Sharples, Yu Jin Kim, David Porter, Kathryn Burns, Soo Hee Jeong, Sarah Benge, Sanjeev Deva, Ben Lawrence, Christopher Jackson, Richard North, R. Matthew Strother, John Duley, Michael Findlay

**Affiliations:** 1 Molecular Medicine and Pathology, Faculty of Medical and Health Sciences, University of Auckland, Auckland, New Zealand; 2 Cancer Trials New Zealand, Faculty of Medical and Health Sciences, University of Auckland, Auckland, New Zealand; 3 Mathematics and Statistics, University of Otago, Dunedin, New Zealand; 4 Auckland City Hospital, Te Whatu Ora Health New Zealand, Auckland, New Zealand; 5 Pharmacology and Clinical Pharmacology, Faculty of Medical and Health Sciences, University of Auckland, Auckland, New Zealand; 6 Canopy Cancer Care, 213 Shakespeare Road, Takapuna, Auckland, New Zealand; 7 Oncology, Faculty of Medical and Health Sciences, University of Auckland, Auckland, New Zealand; 8 Dunedin Hospital, Te Whatu Ora Health New Zealand, Dunedin, New Zealand; 9 Department of Medicine, Otago Medical School, University of Otago, Dunedin, New Zealand; 10 Tauranga Hospital, Te Whatu Ora Health New Zealand, Tauranga, New Zealand; 11 Christchurch Hospital, Te Whatu Ora Health New Zealand, Christchurch, New Zealand; 12 School of Pharmacy and Pharmaceutical Sciences, University of Queensland, Brisbane, Australia

**Keywords:** Thymine, 5-FU toxicity, Dihydropyrimidine dehydrogenase deficiency, DPYD

## Abstract

**Purpose:**

Inherited dihydropyrimidine dehydrogenase (DPD) deficiency is a risk factor for severe 5-fluorouracil toxicity. We report a phenotyping approach (thymine challenge test) to prospectively determine DPD activity and the association with severe adverse events.

**Methods:**

The primary aim of this prospective study was to determine whether a thymine challenge test could prospectively identify patients at risk of severe toxicity from treatment with 5-fluorouracil/capecitabine in combination chemotherapy schedules or monotherapy. The focus was prediction of those at risk of ≥ grade 3 gastrointestinal toxicity. DPD activity was determined from the thymine/dihydrothymine (THY/DHT) ratio measured in a urine sample after a thymine test dose (250 mg, oral).

**Results:**

Of the 166 patients, 11.7% had severe diarrhoea/mucositis. The THY/DHT ratio was not significantly different in these individuals compared to those with minimal toxicity. However, *post hoc* analysis found decreased DPD activity in those who had non-gastrointestinal toxicity, most notably grade ≥ 2 Hand-Foot syndrome (*p* = 0.001).

**Conclusion:**

The data do not support our primary hypothesis that this phenotyping approach would discriminate those at risk of severe/life-threatening gastrointestinal toxicity. The clinical factors which influence gastrointestinal toxicity, particularly in patients receiving CAPOX require further investigation.

**Clinical trial registration:**

ACTRN 12,617,001,109,392 registered 28/07/2017.

**Supplementary Information:**

The online version contains supplementary material available at 10.1007/s00280-025-04804-6.

## Introduction

The use of standard dosing of 5-fluorouracil (5-FU), and its oral prodrug capecitabine, is associated with severe toxicity in approximately 30% of patients. The current consensus is that this toxicity is due to a deficiency in the dihydropyrimidine dehydrogenase (DPD) enzyme, which is responsible for most of the plasma clearance of 5-fluorouracil (5-FU) [1]. This enzyme is encoded by the *DPYD* gene and pre-therapeutic screening of patients for a panel of risk associated SNP has been mandated by the European Medicines Agency (EMA) [2], and the UK Medicines and Healthcare products Regulatory Agency [3]. This panel of risk associated variants (*2A, D949V, *13 & HapB3, defined by rs3918290, rs55886062, rs67376798 and rs56038477) are rare and have a low combined allele frequency. Importantly, this targeted panel does not detect the majority (~ 60–70%) of individuals who experience 5-FU adverse effects. Indeed, the strongest associations are for haematological toxicity, with limited success in identifying those at risk of severe gastrointestinal toxicity [4, 5]. Hospitalisation due to grade 3 or greater diarrhoea and/or mucositis is of clinical concern, especially if a patient also has neutropenic sepsis.

It has been suggested that interrogation of the *DPYD* gene mutation burden based on genome sequencing may identify further risk variants and address this issue [6]. However, recent next generation sequencing of a large cohort has had limited success at identification of additional risk variants [7]. Ultimately it may remain difficult to produce a comprehensive panel of clinically validated risk variants which is comprehensively applicable to all patients receiving 5-FU based chemotherapy. Hence a screening approach which is agnostic to an individual’s specific inherited cause of DPD enzyme deficiency has merit. These phenotype approaches include measurement of 5-FU degradation rate in peripheral blood cells ex vivo, the measurement of endogenous plasma concentrations of uracil, or challenge loading tests with uracil [reviewed in [8]. Thymine is the other endogenous substrate of the DPD enzyme and due to differences in the anabolic and salvage pathways for this pyrimidine, thymine may be a more robust measure of DPD activity than uracil. Indeed, a recent study indicates that it is less prone to post-collection inaccuracies than uracil [9].

The thymine challenge test to phenotype for DPD activity has been established in healthy volunteers [10], and the thymine to dihydrothymine (THY/DHT) ratio was then used in two separate studies to identify patients at risk of severe 5-FU toxicity [11, 12]. We previously conducted a preliminary case-control study [12] focused on patients receiving monotherapy (IV bolus 5-FU or the oral prodrug, capecitabine) that was retrospectively enriched with known toxicity cases. This thymine challenge indicated that individuals with relatively low DPD activity had increased risk of grade 3 or greater (≥ G3) gastrointestinal (GI) toxicity after adjustment for renal function [12]. The sensitivity of the thymine challenge also compared favourably to both plasma uracil concentrations and U/DHU ratios for detection of cases of any grade 3 toxicity [13].

We now report on the THYmine 2 study, which was a prospective observational study to assess the ability of a thymine challenge to identify patients with gastrointestinal or breast cancer who cannot tolerate 5-FU/capecitabine treatment. The primary objective was to determine whether the ratio of THY/DHT measured in a urine sample after a thymine test dose (250 mg, per oral) can discriminate between patients who tolerate 5-FU/capecitabine treatment and those who experience severe 5-FU related gastrointestinal toxicity (diarrhoea or mucositis).

Secondary objectives were (i) to explore the impact of expanding the definition of severe 5-FU toxicity by repeating the primary analysis after adding neutropenia to gastrointestinal toxicity (ii) to determine whether the THY/DHT ratio differs in people who develop clinically significant hand-foot syndrome (grade 2 or 3) and people who tolerate treatment. (iii) to compare the predictive ability of the THY/DHT ratio to the panel of known deleterious SNP in the *DPYD* gene. (iv) to explore the impact of treatment (capecitabine vs. infusional 5-FU) and dosage (total dosage in cycle 1 per body surface area) on the relationship between THY/DHT ratio and diarrhoea and/or mucositis toxicity. (v) to assess inter-individual differences in transmembrane uptake of 5-FU into cells from the buccal mucosa and determine if high uptake is associated with risk of 5-FU toxicity.

## Materials and methods

The clinical trial was registered (ACTRN 12617001109392) and ethical approval of the study was given by Health and Disability Ethics Committee of NZ (17/CEN/149). The study was performed in accordance with the Declaration of Helsinki. Patients were recruited from five sites across New Zealand: Auckland City, Tauranga, Christchurch and Dunedin Hospitals, and Canopy Cancer Care (Milford and Epsom sites) Auckland.

Patients (> 18 years old) able to give written consent and about to receive either 5-FU or capecitabine (as monotherapy or in combination with other agents) for treatment of gastrointestinal cancer or breast cancer (metastatic or residual disease at surgery following neoadjuvant therapy) were recruited into the study. Irinotecan-based combination schedules were excluded, due to the risk of severe diarrhoea from this agent. Other exclusion criteria included prior history of infusional 5-FU or capecitabine treatment, or concurrent abdomino-pelvic radiation therapy. Pregnant or breast-feeding patients were also excluded from the study.

At a pre-chemotherapy clinic visit prior to 5-FU/capecitabine-based treatment all patients received a 250 mg oral dose of thymine. Dosing was undertaken in the morning and participants had eaten their normal breakfast. Patients voided their bladder, and a spot baseline urine sample was collected, immediately prior to dosing. The cumulative urine production was then collected between 0 and 4 h post-dose. The thymine (THY) and dihydrothymine (DHT) concentrations were measured in a 0–4 h cumulative urine sample by LC/MS analysis as previously described [12]. This sample analysis was blinded to the clinical toxicity outcomes. We have previously determined the plasma pharmacokinetics of Thymine and DHT in both healthy volunteers and a case series of patients with severe 5-FU induced toxicity and compared this to the urinary THY/DHT ratio [10, 11]. We have chosen to use a 0–4 h cumulative urine sample to avoid intensive serial blood sampling as this is logistically simpler. The 4 h window of cumulative sample collection covers the period where plasma concentrations of these analytes return to pre-dose baseline.

A baseline blood sample was collected into a 10 mL PAXgene DNA tube (Qiagen). De-identified genomic DNA was screened for following deleterious *DPYD* variants which comprise the EMA recommended risk panel: *2A (rs3918290,c.1905 + 1G > A; IVS14 + 1G), D949V (rs6737698 c.2846 A > T), *13 (rs55886062,c.1679 T > G; I560S), HapB3 (rs56038477, rs75017182, rs56276561) using custom designed Agena MassARRAY iPLEX assays at Grafton Clinical Genomics, Auckland, New Zealand as previously described [12]. Buccal mucosal cells were collected by cytobrush immediately prior to administration of the thymine dose. These samples were assayed for 5-FU uptake [14].

5-FU related adverse events were collected blinded to the THY/DHT ratio and *DPYD* genotype information, using common toxicity criteria for adverse events (CTCAE v4.0). Data were collected for a minimum of 2 cycles (e.g. 8–9 weeks) after the start of 5-FU based treatment. Due to the different length of treatment cycles for each schedule this is approximately 3 cycles of capecitabine and 5 cycles of infusional 5-FU (if there are no dose delays). Participants could contribute information to the primary outcome if they had completed at least one cycle of chemotherapy.

Data were entered into a GCP compliant ALEA database and stored in a secure, ISO 27,001 certified data centre. Controlled access to data entry, audit trails, drop down options, range checks and queries were utilised to ensure the accuracy of the data.

The pre-defined toxicity categories were grade 3 or greater (≥ G3) diarrhoea and/or mucositis requiring hospitalisation, termed “Severe GI toxicity”. An additional category of “Other severe toxicities” which included ≥ G3 neutropenia and/or thrombocytopenia and/or anaemia and/or hyper-bilirubinaemia and/or ≥ G2 Hand-Foot syndrome. The presence of ≥ G3 diarrhoea and/or mucositis super-ceded other toxicities for categorisation. The control category termed “minimal toxicity” was classed as the absence of the above toxicities at those grades.

### Statistical analysis

Analyses were undertaken based on the predefined statistical plan. Due to the skewed distribution of the THY/DHT ratios within the cohort, descriptive data are reported as median and interquartile range (IQR). Deviation from a normal Gaussian or log-normal distribution was confirmed visually with QQ plots and the Shapiro-Wilk test [15]. Non-parametric statistical tests were used to compare distributions, the Wilcoxon rank sum test for comparisons of two groups and Kruskal-Wallis tests for pairwise comparisons [16]. Sensitivity of a THY/DHT ratio test for severe GI toxicity was calculated for a cut-point giving a specificity of 85%, and the proportion and an exact 95% confidence interval are presented. Renal function (eGFR) was approximately normally distributed, and group means were compared using ANOVA. Logistic regression was used to explore the impact of chemotherapy regimen, dose adjustment for body surface area and renal function on the relationship between THY/DHT ratio and severe GI toxicity. Association of *DPYD* variant status and toxicity used Fishers exact test. Agreement between the *DPYD* SNP based test and the THY/DHT ratio was evaluated using a Kappa statistic. Two-sided p-values < 0.05 were considered statistically significant. Primary analyses were undertaken in Stata and R [17, 18]. Graphs were produced using GraphPad Prism v10.

Sample size calculations were based on an estimated incidence of 25% grade 3 or greater (≥ G3) diarrhoea, mucositis or neutropenia (DMN), including an 8% incidence of grade 4 or greater (≥ G4) of these events. We aimed to recruit 200 patients, anticipating that 50 would experience these ≥ G3 toxicities. This would give 80% power to detect a standardised difference in means between the severe and minimal toxicity groups of 0.5, if the variances in the two groups are equal. For life-threatening ≥ G4 diarrhoea, mucositis or neutropenia toxicity, we anticipated 16 patients, which would give 80% power to detect a standardised difference of 0.9. A change was made to the protocol, prior to analysis, to consider only diarrhoea, mucositis (GI) toxicity in the primary objective. For this, the study will have 80% power to detect a standardised difference in means of 0.8 if GI toxicity accounts for half the ≥ G3 DMN toxicities.

## Results

**Fig. 1:**
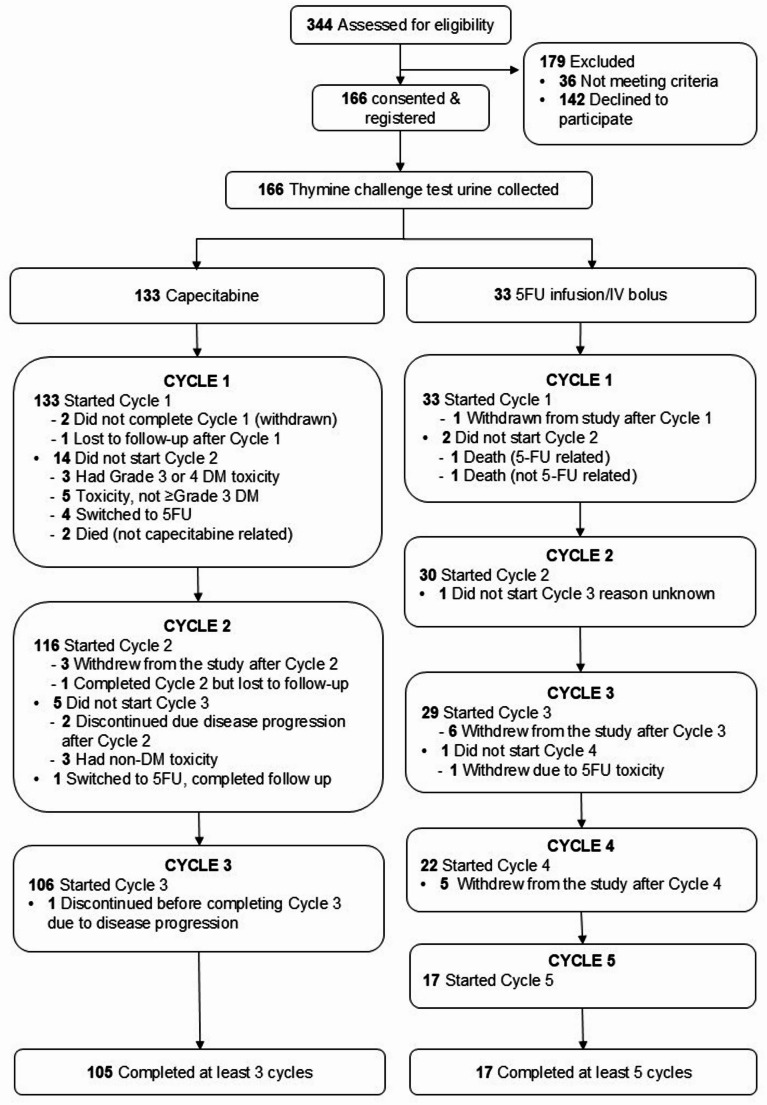
Flow of participants through the study (CONSORT diagram). Participants were recruited between 23rd November 2017 and 19th January 2022 from 5 sites across New Zealand (Auckland City, Tauranga, Christchurch and Dunedin Hospitals and Canopy Cancer Care, Auckland). DM: diarrhoea or mucositis

Planned recruitment was 200, however due to the impact of COVID-19 restrictions only 166 participants were recruited. Recruitment and retention are illustrated in Fig. 1. The demographic information (age, biological sex and self-declared ethnicity as well as ECOG performance status) are shown in Supplementary Table 1.

All patients completed the thymine challenge test. The ratio of THY/DHT was not normally distributed in this cohort (*p* < 0.0001) and did not fit a log-normal distribution (*p* < 0.001). The median THY/DHT ratio in the cohort was 2.3 (IQR 1.1–4.3) and was not significantly different from the data previously collected in our preliminary study [12]. The scatterplot of the distribution of the THY/DHT ratios (Supplementary Fig. 1) demonstrates the skew of the data, suggestive of a multi-modal distribution, typical of a pharmacogenetic trait. Plotting the data on a logarithmic axis allows visualisation of the 39 participants with values greater than the interquartile range (individuals with low DPD activity) and importantly also the 35 patients who had values lower than the interquartile range. This latter group is suggestive of an ultra-fast metaboliser trait.

The majority (*n* = 130) of the participants received capecitabine, either as monotherapy or in combination with oxaliplatin (CAPOX). Details of the chemotherapy regimens received are provided (Supplementary Table 2). Three patients received a regimen containing irinotecan, which was an exclusion criterion, however these participants are included in the primary data analysis following sensitivity analyses which determined there was minimal impact on the results.

The median follow-up was 63 (IQR, 60–80) days. Three participants withdrew from the study early during cycle 1 and did not have sufficient on study exposure to 5-FU/capecitabine to provide information on toxicity response to treatment and are excluded from analyses. Hence there were 163 participants available to determine the relationship between thymine challenge test and 5-FU related toxicity. These adverse events are shown in Table 1.

**Table 1:** The prevalence of 5-FU induced adverse events and the relationship to dihydropyrimidine dehydrogenase activity (THY/DHT ratio)

	Category	*n*	%	THY/DHT ratio Median (IQR)	
	**Minimal toxicity** (none of the events listed below)	**108**	**66.3**	1.79 (0.97, 4.09)	Reference (Comparator Group)
	**Severe gastrointestinaI (GI) toxicity (DM)** ^a^	**19**	**11.7**	2.12 (0.81, 2.99)	*p* = 0.83^c^
	Diarrhoea ≥ G3 (*n* = 15)				
	Mucositis ≥ G3 (*n* = 4)				
	**Other severe toxicity**	**36**	**22.0**	3.39 (2.30, 5.47)	*p* = 0.025^d^
	Neutropenia ≥ G3 (*n* = 13)				
	Thrombocytopenia ≥ G3 (*n* = 3)				
	Anaemia ≥ G3 (*n* = 0)				
	Hyperbilirubinemia ≥ G3 (*n* = 2)				
	Hand-foot syndrome ≥ G2^b^(*n* = 20)				
Secondary objective	**≥G3 diarrhoea**,** mucositis or neutropenia (DMN)**	**30**	**18.4**	2.49 (1.11, 3.21)	*p* = 0.3^c^
*Post-hoc* analysis	**Hand-foot syndrome (≥ G2)** ^b^	**20**	**12.1**	4.32 (2.53, 5.47)	*p* = 0.001^c^

Most patients did not experience multiple severe toxicities simultaneously, when this occurred diarrhoea/mucositis (DM) was prioritized for categorisation purposes. Minimal is the absence (or lower grade) of the other categories of toxicity. ^a^ Hospitalised with this toxicity. ^b^This includes 8 participants with G3 toxicity. ^c^p-value from Wilcoxon rank sum test comparing THY/DHT distribution to that for Minimal toxicity. ^d^Kruskal Wallis test comparing distributions of THY/DHT in the 3 toxicity categories (Minimal, Severe GI (DM) toxicity and Other severe toxicity) gave *p* = 0.0011. Pairwise comparison of Other severe toxicity group to Minimal toxicity group (*p* = 0.025)

The prevalence of any ≥ G3 toxicity was 22.7%, consistent with the literature incidence. Most of the patients did not experience multiple severe different toxicities simultaneously. The three participants with G3 oral mucositis had ≤ G2 diarrhoea. The majority (93%) of those with severe gastrointestinal (GI) toxicity had non-neutropenic diarrhoea. Of the patients with severe GI toxicity, hospitalisation ranged between 1 and 29 days (median 6.5 days). Four patients experienced life-threatening (G4) diarrhoea and were hospitalised for 7–28 days (median, 18 days).

Four patients changed from capecitabine to 5-FU after the first cycle and one after the second cycle (due to nausea, hand-foot syndrome (G2), cardiac toxicity or diarrhoea (G3), and one patient with reaction to oxaliplatin infusion). Dose adjustment due to toxicity occurred in 35 participants receiving capecitabine and 10 receiving 5-FU (infusional or IV bolus). There were 28 patients who discontinued treatment early due to drug-related adverse events, 11 of these individuals had a prior dose adjustment. Eight patients receiving infusional 5-FU and 8 patients receiving capecitabine-based treatment had dose delays (> 4 days) due to drug-related adverse events.

### Primary objective

**Fig. 2:**
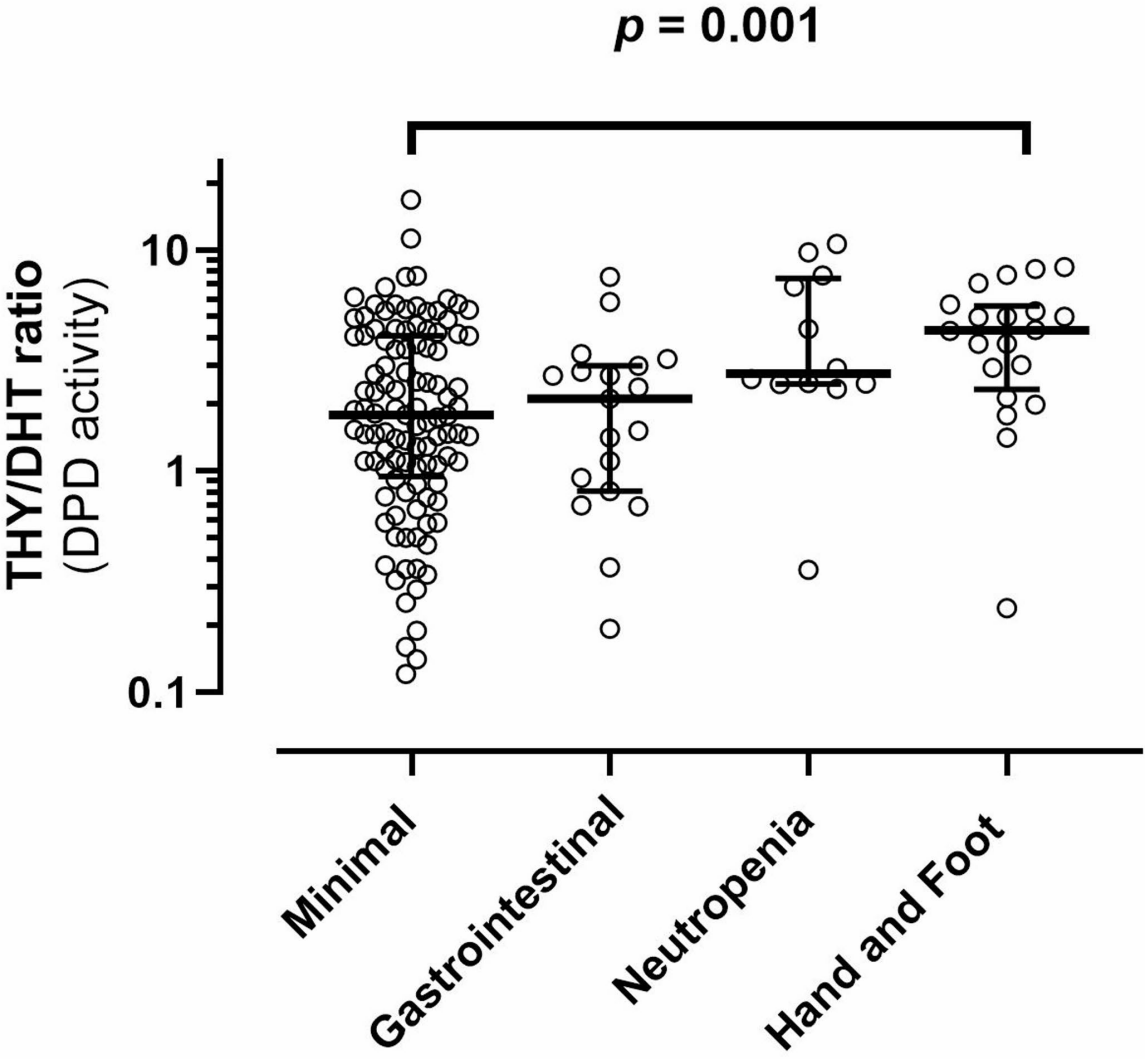
Dihydropyrimidine dehydrogenase (DPD) activity appears to be decreased in patients with clinically severe Hand-Foot syndrome (≥ G2) compared to those with Minimal toxicity. Gastrointestinal toxicity (≥ G3 diarrhoea/mucositis); Neutropenia (≥ G3); Hand-Foot syndrome (≥ G2). Minimal (absence of these adverse events at these grades). THY/DHT ratios are shown on a log-scale for clarity due to the substantial positive skew of the data. Solid line indicates the median (IQR) of the data. Severe gastrointestinal toxicity was prioritised over other toxicities in coding. Three participants with severe gastrointestinal toxicity also had grade 2 Hand-Foot syndrome; One patient with grade 4 diarrhoea also had grade 3 neutropenia, and one patient with grade 3 mucositis had grade 3 neutropenia. p- value is the statistical difference between minimal and Hand-Foot toxicity groups

The primary objective was to determine whether the ratio of THY/DHT can discriminate between patients who experience minimal 5-FU related toxicity (defined as absence of any other severe (≥ G3) haematological, liver or ≥ G2 skin toxicity) and those who experience severe (≥ G3) diarrhoea/mucositis (DM) related toxicity and were hospitalised. Overall, 108 (66.3%) experienced minimal toxicity (Table 1) and these are the comparator group as pre-specified in the analysis plan. Nineteen (11.7%) patients experienced ≥ G3 DM toxicity, but there was no statistically significant difference (*p* = 0.83), between the distribution of the THY/DHT ratio in these patients (2.12 IQR 0.81–2.99) and the comparator group (1.79 IQR 0.97–4.09), (Fig. 2). Furthermore, the subgroup of four patients with life-threatening diarrhoea unexpectedly had relatively high DPD activity, with low THY/DHT ratio ranging 0.194–2.81 (median 1.31). Small numbers precluded a separate statistical comparison of the THY/DHT ratio in those with life-threatening DM toxicity compared with minimal toxicity.

Analysis of the sensitivity of the THY/DHT ratio to identify those with severe or life-threatening DM toxicity was undertaken, the cut-off (5.003) was determined by fixing the specificity at 85% (pre-specified in the analysis plan). With this cut-off value, 28 participants were classified as having a positive THY/DHT ratio, and the sensitivity to detect ≥ G3 DM toxicity was poor at 10.5% (95% CI, 0.0–24.3).

### Secondary objectives

(i) Repeating the primary analysis including ≥ G3 neutropenia gave 30 (18.4%) participants with severe or life-threatening DMN toxicity, and the median (IQR) for THY/DHT ratio was 2.49 (1.11, 3.21). This distribution did not significantly differ from the comparator group with minimal toxicity (*p* = 0.3). (ii) A *post hoc* analysis of the DPD activity in patients with non-GI toxicities (Fig. 2) is suggestive of a trend to lower DPD activity (elevated THY/DHT ratios) in patients with either neutropenia or Hand-foot syndrome toxicities. This was only statistically significant for clinically severe (≥ G2) Hand-Foot syndrome compared to the Minimal toxicity category (Table 1). When analysed as the predefined “Other severe toxicity” category these individuals had significantly decreased DPD activity (*p* = 0.025) compared to the Minimal toxicity category (Table 1).”
(ii) Comparing the Thymine challenge to the *DPYD* SNP panel: *DPYD* status was unknown for one participant. Seven individuals in the cohort (4.2%) were positive for one of the panel of *DPYD* risk variants. All other patients (158) did not test positive for any of these variants. No SNP deviated from Hardy-Weinberg equilibrium. The observed minor allele frequency (MAF) for each SNP were as follows: rs3918290 MAF 0.00303; rs56038477, rs75017182 and rs56276561 MAF 0.01818 (i.e. complete concordance for all three of the SNP in this haplotype). Neither rs55886062 nor rs67376798 were observed in this cohort. Based on the self-identified ethnicity of the participants (Supplementary Table 1) the majority (78.3%) of the patients were of European ancestry. In this cohort of 165 individuals, these rare *DPYD* variants occur at a prevalence similar to the expected MAF reported for European populations in the dbSNP database (www.ncbi.nlm.nih.gov/snp). The expected prevalence of these variants in people of Māori or Pacific ancestry (11.4% of participants) is currently unknown. In the seven heterozygous carriers the THY/DHT ratio ranged between 0.12 and 4.9, with a median value of 2.9. This was not significantly different than the values for those (*n* = 159) who were homozygous wildtype (reference allele) for each of these risk variants, with median THY/DHT ratio 2.2 (min-max range 0.051-17). Of the seven variant heterozygote carriers, six were positive for the intronic variant rs75017182 (HapB3) with median THY/DHT ratio of 3.06 (range 0.12–4.9). The one individual who was a carrier of *2A variant had a THY/DHT ratio of 1.11. Chemotherapy was not dose-adjusted in these patients based on genotype since this was not part of recommended clinical practice at the time of recruitment into the study. Agreement between the THY/DHT test and genotype was poor (kappa = 0.019, two-sided *p* = 0.22). There was also no clear association between *DPYD* genotype and toxicity. *DPYD* variant carriers and those wildtype for these risk variants in each toxicity category were as follows: Minimal toxicity (*n* = 3 vs. *n* = 104), Severe GI toxicity (*n* = 2 vs. *n* = 17), Other severe toxicities (*n* = 2 vs. *n* = 34). However, the individual who was a *2A heterozygote carrier required a 75% dose decrease (CAPOX schedule) at cycle 2 following G4 diarrhoea, G2 mucositis and G3 neutropenia at cycle 1 (hospitalisation for 7 days). Of the six patients who tested positive for HapB3, three required a dose decrease (25–35%) and one switched therapy from CAPOX to mFOLFOX6. Three (50%) of the HapB3 carriers were categorised as ‘Minimal toxicity’ based on our pre-defined criteria. Supplementary Table 3 provides details of the toxicities observed in these variant carriers. Given the very low sensitivity of the THY/DHT ratio test and the small numbers we did not undertake modelling to determine whether the THY/DHT ratio test provided additional capacity for risk prediction to a SNP-based test.

(iv) Impact of treatment type and dosage on DPD activity and toxicity: Of the 129 participants on capecitabine, 15 (11.6%) experienced a severe DM toxicity, compared with 3 of 32 (9.4%) on 5-FU. Severe DM toxicity was highest for participants on CAPOX (12 of 61, 19.7%), those on capecitabine monotherapy mostly experienced ≥ G2 Hand-Foot syndrome (15 of 64, 23.4%). Those on FOLFOX6 predominantly experienced severe neutropenia (8 of 21, 38.1%). Distributions of THY/DHT ratios by regimen and toxicity are shown in Supplementary Fig. 2. Logistic regression analysis found no evidence that the relationship between THY/DHT ratios and DM toxicity differed between capecitabine vs. 5-FU infusional regimens (interaction *p* = 0.97). There was no evidence that adjustment for body surface area affected the relationship (interaction *p* = 0.99).
(v) Transmembrane uptake of 5-FU into cells from the buccal mucosa: The distribution of transmembrane uptake of 5-FU into buccal mucosa cells was highly positively skewed, with a median (IQR) of 14.6 (4.9, 46.7) pmol/min/10^5^ live cells. Those with severe DM toxicity tended to have lower uptake that those with minimal toxicity with median (IQR) 5.2 (3.6, 15.9) and 14.9 (5.4, 49.0) pmol/min/10^5^ live cells respectively, however the difference was not statistically significant (*p* = 0.2).

### Additional unplanned analyses

Cardiac adverse events (not graded) were noted for 11 patients (6.7%) including one fatality. That individual was not variant for any of the *DPYD* risk alleles tested and the THY/DHT ratio was 2.30, which was close to the median value of the whole cohort.

Our previous preliminary study [12] had focussed solely on patients who received monotherapy (capecitabine or IV bolus 5-FU). Hence, we undertook a *post-hoc* analysis to see if there was any relationship between toxicity profile for type of chemotherapy regimen (monotherapy or combination chemotherapy) and THY/DHT ratio. There was higher proportion of GI toxicity (≥ G3 diarrhoea and/or mucositis) in those receiving CAPOX, whereas those patients receiving mFOLFOX6 predominantly experienced severe neutropenia (Supplementary Fig. 3). Patients receiving FLOT, IV bolus 5-FU (Mayo regimen), mFOLFOX6 without IV bolus 5-FU, or capecitabine plus cisplatin/gemcitabine (PDXG) are not included in this analysis due to the small number on each individual regimen (total *n* = 9).

For capecitabine monotherapy the two individuals with severe GI toxicity had relatively slow DPD activity (THY/DHT ratios 5.8 and 7.55) compared with the median (IQR) values of the minimal toxicity category 1.8 (0.84–4.1). In contrast, patients receiving capecitabine in combination with oxaliplatin (CAPOX), the median THY/DHT ratio in those with severe GI toxicity was lower than that observed in the minimal toxicity group; median (IQR) 1.1 (0.69–2.7) versus 1.8 (0.6–4.3) respectively. There was no difference in THY/DHT ratio between the severe GI toxicity and minimal toxicity group for patients receiving infusional 5-FU in combination with oxaliplatin (mFOLFOX6) with a median (IQR) of 2.6 (2.1-3.0) versus 2.5 (1.3–3.2). However, elevated THY/DHT ratios (decreased DPD activity) were observed for ≥ G2 Hand-Foot syndrome compared to minimal toxicity for both capecitabine monotherapy and combination therapy (CAPOX).

Since poor renal function is known to associate with increased risk of toxicity the eGFR (which is adjusted for age and sex) was calculated from the baseline serum creatinine prior to initiation of treatment (data available for 161 participants). The eGFR values ranged from 35.9 to 121.7 mL/min/1.73m^2^ (mean ± SD, 87.55 ± 17.03 mL/min/1.73m^2^). There was no relationship between toxicity category and renal function (Supplementary Table 4). Inclusion of renal function as a covariate did not improve the ability of DPD phenotype to detect risk of severe GI (DM) toxicity.

## Discussion

We have used a thymine-based challenge test to phenotype for DPD activity with detection of the parent compound and metabolite in urine samples. This is the classical approach used in diagnosis of inborn errors of metabolism. Use of urine sampling also avoids the post-collection conversion due to enzyme activity in blood cells that can compromise the use of endogenous plasma uracil testing. This test also measures the total DPD activity in all organs and tissues and does not rely on the activity in isolated PBMC as a surrogate marker of liver enzyme activity. The thymine challenge dosing was undertaken mid-morning after consumption of breakfast and allows assessment of a ‘snapshot’ of DPD function in this cohort at this time of day. This should minimise any possible effect of diurnal changes in DPD activity on the range of activities we observe across this cohort. This has enabled us to undertake an extensive survey of inter-individual differences in DPD activity in cancer patients following a pyrimidine challenge test. There was a multimodal distribution of DPD activity in this cohort of cancer patients, which is a classical feature of a pharmacogenetic trait [19]. Whilst we did not observe any individuals with complete DPD deficiency (theoretical THY/DHT > 100), there was evidence of a substantial proportion of patients with a very low metabolic ratio and hence a previously unrecognised ‘ultrarapid’ DPD phenotype. These individuals may be at increased risk of therapeutic failure and this phenotype could account for the substantial number of patients who have higher clearance of 5-FU and are underdosed when BSA-based dosing is used [20]. Indeed, there have been reports that high DPD activity (measured in PBMC) associates with substantially worse overall survival and progression free survival [21]. This “high activity” phenotype may relate to the recently identified gain of function (rs4294451) *DPYD* variant [22].

Our previous publications using this thymine challenge in a case series compared to healthy controls [11] suggested that a THY/DHT ratio of > 4 (suggestive of partial DPD deficiency) might be associated with increased risk of severe/life-threatening 5-FU related toxicity. We then undertook a small case-control study in patients receiving monotherapy to address whether this test could identify individuals at risk of severe GI toxicity (diarrhoea and/or mucositis) [12],. We focused on GI toxicity, since neutropenia can be asymptomatic and options for clinical management are well developed. Indeed, some combination schedules, such as FLOT, include prophylactic pegfilgrastim due to the relatively high incidence of neutropenia in this schedule. In contrast, the severe enteropathy induced by 5-FU often results in prolonged hospitalisation and hence is of substantial clinical concern. The prevalence of the “global” severe (≥ G3) toxicity (i.e. haematological, Hand-Foot Syndrome, diarrhoea/mucositis) that we observed in the current prospective study (25.7%) is similar to that previously reported by many authors prior to adoption of genotype-guided dosing [23, 24].

Our data do not support our primary hypothesis that the thymine challenge test (THY/DHT ratio) would prospectively discriminate those at risk of severe/life threatening GI toxicity from those with minimal toxicity. However, we did find significantly decreased DPD activity in those at risk of non-GI toxicity, most notably grade 2 or greater Hand-Foot syndrome. However, many patients with elevated THY/DHT ratios (indicative of low DPD activity) did not experience substantial toxicity of any type. Additional factors which could influence risk of toxicity may include dietary folate status. The inclusion of folinic acid as part of IV/infusional 5-FU regimens increases the tumour response, and folate status is also known to increase normal tissue toxicity [25]. Moreover, the use of multivitamins, which contain folates, has been shown to precipitate severe GI as well as Hand-Foot toxicity in patients receiving capecitabine [26]. Hence the dietary folate status of patients remains an underexplored environmental factor, additional to DPD activity, that may influence risk of severe toxicity.

It is well established that different schedules and regimens of 5-FU/capecitabine associate with different types of normal tissue toxicity. For example, IV bolus 5-FU associates with severe neutropenia [27], whereas capecitabine monotherapy (BID) associates with a higher incidence of Hand-Foot syndrome [28]. Our analysis of the relationship between DPD activity and severe adverse events in patients receiving different schedules further highlights the important consideration of drug regimen as well as DPD status on adverse outcomes, as recently noted by others [29]. The use of capecitabine may further complicate associations between DPD activity and 5-FU induced toxicity, due to variation in the multiple-step activation of this prodrug formulation into 5-FU.

Importantly, the high prevalence (19.6%) of severe diarrhoea in patients receiving CAPOX, which was not associated with elevated THY/DHT ratios, suggests factors other than DPD deficiency are likely to be important. This could relate to pharmacological factors which, in addition to the effect of oxaliplatin include, the 5-FU steady state plasma concentrations achieved following different doses in different regimens. In addition, after capecitabine administration the concentration of 5-FU in normal colon tissue is 8-times higher than in plasma. Whereas, after either IV bolus or infusional 5-FU the concentration in the colon tissue was similar to that in plasma [30].

It is also possible that in addition to inherited DPD deficiency, phenoconversion may also occur. For example, administration of capecitabine prodrug (BID) daily over 14 days, leads to a 60% increase in plasma 5-FU AUC by day 14 [31]. In addition, following IV bolus and infusional dosing there is a time-dependent decrease in 5-FU clearance [32, 33] this occurs despite the short-half-life of 5-FU. This suggests some form of autoinhibition of DPD activity, possibly via *DPYD* gene down-regulation. The possible effect of oxaliplatin on DPD activity has also been explored [34] and remains unclear, with possible delayed effects on the pharmacokinetics of 5-FU postulated [35, 36]. Importantly there have been no studies investigating possible delayed effect of oxaliplatin on 5-FU pharmacokinetics following BID dosing of capecitabine for 14 days.

It is notable that the seven individuals who tested positive for one of the validated panel of *DPYD* risk variants did not have elevated THY/DHT ratios compared those who were wildtype at these alleles. Indeed, many other studies investigating the relationship between the EMA panel of *DPYD* risk variants and other endogenous phenotypic biomarkers of DPD activity also note substantial overlap with those who are wildtype for these variants. For example, the correlation between *DPYD* genotype and plasma uracil levels is also weak (r^2^ = 0.15) [37],. This is likely due to the currently unidentified factors which result in relatively low DPD activity in a substantial proportion of people. Moreover, in heterozygote carriers the influence of the other allele on the overall activity of the enzyme product is unknown. This is particularly pertinent because using the thymine phenotyping approach, we have identified a possible “ultra-rapid metaboliser” group with very high DPD activity. This could account for why only 50% of DPYD*2A carriers have severe toxicity and only 41% of HapB3 heterozygotes have 5-FU associated toxicity when receiving combination chemotherapy for colon cancer [38]. Our findings (in a much smaller cohort) reiterate this poor association between HapB3 status and severe toxicity. This raises the concern about underdosing of patients if *DPYD* genotyping does not always identify a functional loss of enzyme activity.

Although DPD is considered to be the major contributor to the plasma elimination of 5-FU, renal clearance also plays a role [39]. We previously demonstrated a significant association between renal function and GI toxicity (when eGFR was calculated using the Cockcroft Gault formula) in our preliminary case-control study. Using that method of determining renal function as a covariate improved the ability of the THY/DHT ratio to discriminate cases from non-cases. Others have also reported associations between renal function and toxicity [29, 40, 41]. In the current study we did not observe any association between eGFR and toxicity. However, the Cockcroft Gault formula has inaccuracies particularly in high or low BMI individuals, the use of the CKD-EPI equation in the current study may explain this discrepant outcome. Moreover, it is important to note, that age and sex have been reported to associate with toxicity outcomes and differences in renal function are influenced by both of these intrinsic factors.

In summary, the thymine challenge test to phenotype for DPD activity has demonstrated substantial inter-individual differences in activity, including a previously under-appreciated ‘ultrarapid metaboliser group”. Whilst relatively low DPD activity associated with Hand-Foot syndrome there was no association with severe/life-threatening GI toxicity. The clinical factors which influence GI toxicity risk, particularly in those patients receiving CAPOX schedule require further investigation. However, recent reports suggest an intriguing role of DPD metabolism by the gut microbiome [42–44] as well as the influence of antibiotics on toxicity and effectiveness of FOLFOX regimens [45]. The role of the microbiome could be a further confounding factor in the ability to predict GI toxicity risk following use of 5-FU/capecitabine.

## Supplementary Information

Below is the link to the electronic supplementary material.

Supplementary Material 1

## Data Availability

All data supporting the findings of this study are available within the paper and its Supplementary Material.
