# Supplementary material for: A thymine-challenge test to prospectively evaluate dihydropyrimidine dehydrogenase activity for risk of severe 5-fluorouracil-induced gastrointestinal toxicity
Source: Cancer Chemother Pharmacol. 2025 Aug 18;95(1):81. doi: 10.1007/s00280-025-04804-6 (PMC12361283; doi:10.1007/s00280-025-04804-6)
Supplement: Supplementary file 1 — Supplementary Material 1 [file 280_2025_4804_MOESM1_ESM.pdf]

Supplementary material

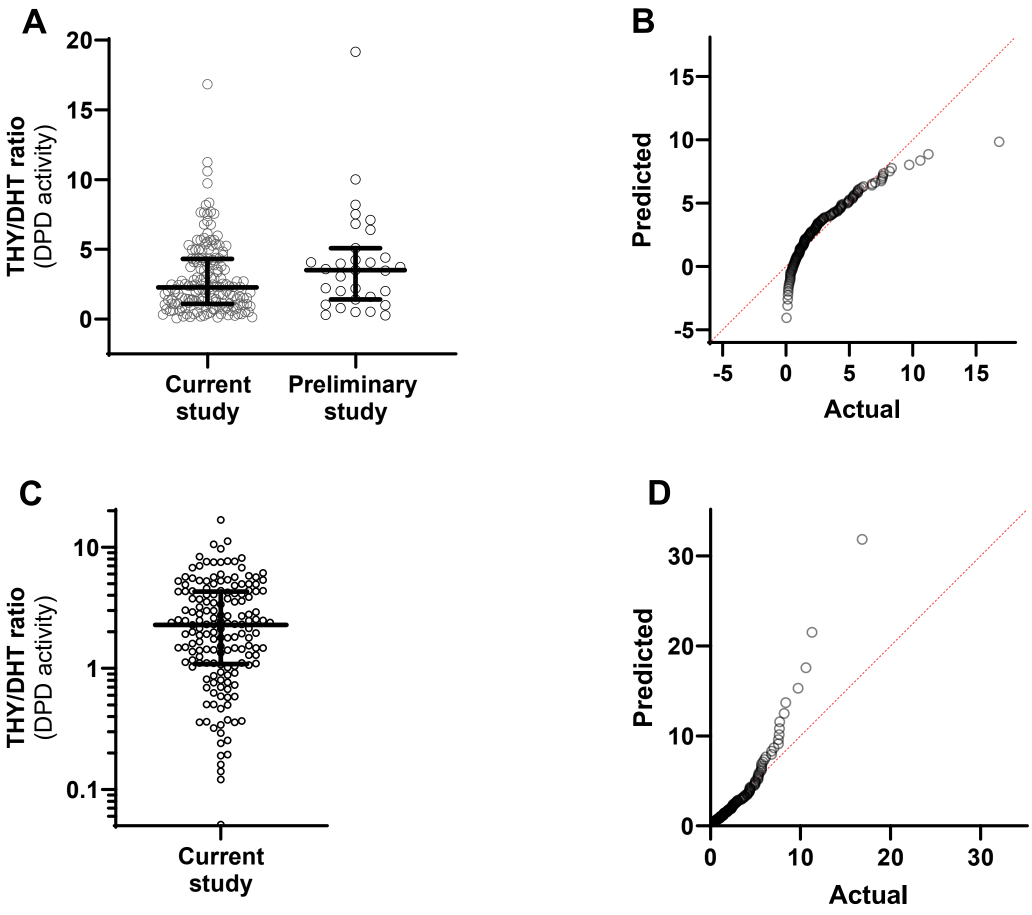

Supplementary Figure 1: The range of THY/DHT values is suggestive of substantial inter-individual differences in dihydropyrimidine dehydrogenase (DPD) activity across the patient group.
A) Scatterplots of the range of THY/DHT values observed in the current study cohort in comparison to the previously reported preliminary data[a(12)]. Median and IQR are shown. There was no statistically significant difference (p=0.1274, two-tailed Mann Whitney test) in the THY/DHT ratio between the two cohorts.
B) Normal QQ plot of the current study provides a visual indication of how the data deviate from a Gaussian distribution, with both negative and positive skew observed.
C) The THY/DHT ratio data are plotted on a logarithmic scale to aid visualisation of the proportion of 'ultra-rapid metabolisers" with values below the IQR as well as those with relatively slow DPD activity, values above the IQR.
D) Log-normal QQ plot is a visual indication that the data also significantly (p <0.001) deviate from a log-normal distribution, which is further suggestive of more than one phenotypic trait in the population.
[a] Prospectively collected patients only (n= 31).

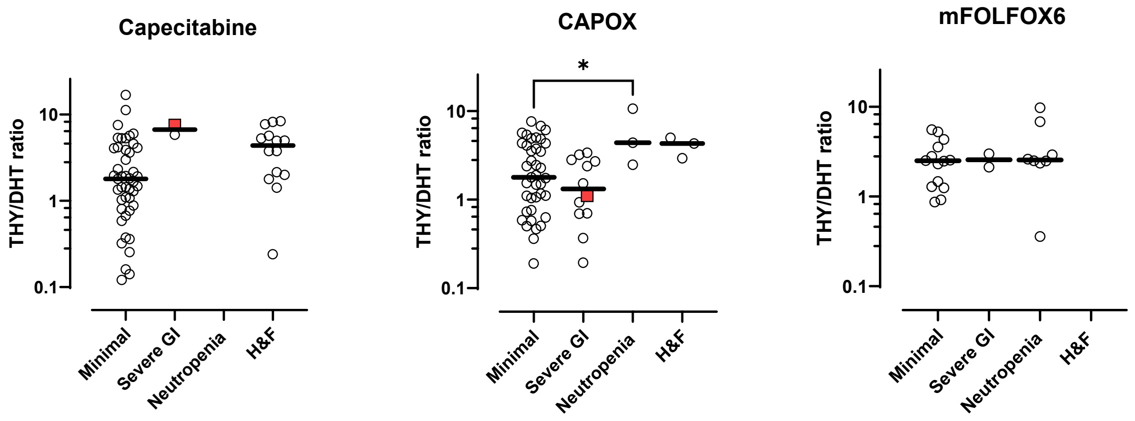

Supplementary Figure 2: Dihydropyrimidine dehydrogenase activity (THY/DHT ratios) and toxicity in relation to the three main treatment regimens used in this cohort.

Capecitabine 21 days* BID 1250 mg/m^2 or *continuous for breast cancer; CAPOX 14 days capecitabine BID 1000 mg/m^2 and d1 oxaliplatin IV 130 mg/m^2; mFOLFOX6 d1 oxaliplatin IV 85 mg/m^2, 5-FU IV bolus (400 mg/m^2) + 2 days infusion 2400 mg/m^2 and folinate. Solid (red) square symbols indicate the two individuals who had severe neutropenia as well as severe GI toxicity. * $p < 0.05$, one way-ANOVA.

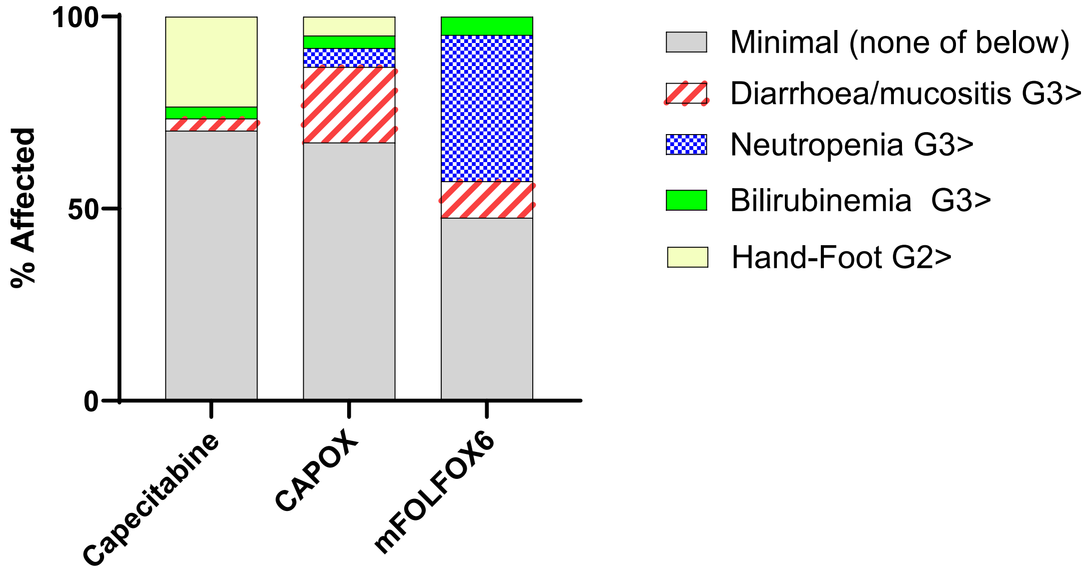

Supplementary Figure 3: The incidence of toxicity categories relative to the chemotherapy regimen.

Supplementary Table 1: Demographic and clinical characteristics of participants.

		N	%
Age	30-39	9	5.4
	40-49	22	13.3
	50-59	38	22.9
	60-69	49	29.5
	70-79	42	25.3
	80-89	6	3.6
Biological sex	Female	99	59.6
	Male	67	40.4
Ethnicity[1]	European	130	78.3
	Maori	10	6.0
	Pacific	9	5.4
	Asian	9	5.4
	MELAA	2	1.2
	Other	6	3.6
Performance status (ECOG)	0	125	75.3
	1	35	21.1
	2	4	2.4
	Missing	1	0.6
	Not Done	1	0.6
Cancer type	Biliary	3	1.8
	Gastro-oesophageal	6	3.6
	Pancreatic	5	3.0
	Small bowel	4	2.4
	Colon	87	52.4
	Rectal	8	4.8
	Metastatic breast	43	25.9
	Non-metastatic breast	6	3.6
	Appendix	3	1.8
	Gallbladder	1	0.6

[1] Self-declared ethnicity based on NZ census categories. MELAA: Middle Eastern, Latin American or African

Supplementary Table 2: Chemotherapy schedules received at cycle 1 and details of each regimen

Regimen	Number
**Capecitabine monotherapy**	**65** (46 breast cancer)
**CAPOX**	**61**
**Other capecitabine** (PDXG)	**4**
**mFOLFOX6**	**21**
**Other 5-FU schedules**	**12**
mFOLFOX no IV bolus	4
mFOLFOX no LV	2
MAYO (FU/FA)	2
FLOT	1
FOLFIRI	2
FOLFIRI + cetuximab	1
Total	**163**

Capecitabine

Drug	dose	route	day	
	1250 mg/m^2 Twice daily	oral administration	1 to 14#	#Continuous for metastatic breast cancer

CAPOX

Drug	dose	route	day	
oxaliplatin	130 mg/m^2	intravenous	1	120 minutes
capecitabine	1000 mg/m^2 Twice daily	oral administration	1 to 14	

mFOLFOX6

Drug	dose	route	day	
oxaliplatin	85 mg/m^2	intravenous	1	120 minutes
foliNIc acid	400 mg/m^2 or 50 mg flat dosing	intravenous	1	120 minutes or 2 minutes
fluorouracil	400 mg/m^2	intravenous	1	15 minutes
fluorouracil	2400 mg/m^2	intravenous	1	46 hours

Bolus 5-FU (Mayo regimen)

Drug	dose	route	day	
foliNIc acid	20 mg/m^2	intravenous	1-5	Slow bolus
fluorouracil	425 mg/m^2	intravenous	1-5	Slow bolus

FLOT

Drug	dose	route	day	
DOCEtaxel	50 mg/m^2	intravenous	1	60 minutes
oxaliplatin	85 mg/m^2	intravenous	1	120 minutes
foliNIc acid (as calcium folinate)	200 mg/m^2 Or 50 mg flat dosing	intravenous	1	120 minutes or 2 minutes
fluorouracil	2600 mg/m^2	intravenous	1	24 hours
pegFILGRASTIM	6 mg	subcutaneous injection	3	

PDXG

Drug	dose	route	day	
DOCEtaxel	25 mg/m^2	intravenous	1	60 minutes
gemcitabine	800 mg/m^2	intravenous	1	30 minutes
cISplatin	30 mg/m^2	intravenous	1	60 minutes
capecitabine	625 mg/m^2 Twice daily	oral administration	1 to 14	

Supplementary Table 3: ***DPYD*** **risk variant carriers and toxicity outcomes.**

Risk variant	Toxicities and CTCAE grade (G)	Days hospitalised	THY ratio	eGFR ml/min/1.73m^2	Regimen	Cancer type	Comments
*2A	G4 Diarrhoea<br>G2 Mucositis<br>G3 Neutropenia<br>G1 Anaemia	7	1.108	74.98	CAPOX	Colon	75% dose decrease at cycle 2
HapB3	G4 Neutropenia<br>G2 Thrombocytopenia<br>G1 Anaemia	0	2.91	50.98	mFOLFOX6	Upper GI	Dose decrease (25%) at cycle 4<br>8-day dose delay
HapB3	G3 Diarrhoea<br>G1 Hand-Foot<br>G1 Neutropenia<br>G1 Anaemia	18	3.20	91.53	CAPOX	Small bowel	25% dose decrease at cycle 2 then discontinued
HapB3	G2 Hand-Foot<br>G1 Diarrhoea	0	3.76	101.55	Capecitabine	Metastatic Breast	25% dose decrease cycle 2, further dose decrease at cycle 3.<br>Total dose decrease = 35%<br>4-week dose delay then discontinued
HapB3	G2 Diarrhoea<br>G1 Neutropenia<br>G2 Anaemia	5	4.91	88.12	CAPOX	Colon	2 cycles only, patient request to cease treatment
HapB3	G2 Diarrhoea<br>G2 Mucositis<br>G1 Neutropenia	0	0.627	91.92	CAPOX	Colon	Changed to mFOLFOX6 at 25% dose decrease
HapB3	G0<br>No toxicities observed	0	0.12	118.26	Capecitabine	Metastatic Breast	

Supplementary Table 4: Descriptive statistics of renal function (eGFR) relative to toxicity category.

	n	**eGFR (mL/min/1.73m^2)** Mean ±SD
Minimal toxicity	106	87.55 ± 17.12
Severe GI toxicity	19	82.35 ± 15.34
Other severe toxicities	36	90.82 ± 15.34

eGFR was calculated using the following equations:

	**Creatinine**	
FEMALE	≤ 62 micromol/L	$eGFR_{CKD-EPI}$ = 144 × (SCr × 0.0113/0.7)-0.329× (0.993)age
	> 62 micromol/L	$eGFR_{CKD-EPI}$ = 144 × (SCr × 0.0113/0.7)-1.209 × (0.993)age
MALE	≤ 80 micromol/L	$eGFR_{CKD-EPI}$ = 141 × (SCr × 0.0113/0.9)-0.411 × (0.993)age
	> 80 micromol/L	$eGFR_{CKD-EPI}$ = 141 × (SCr × 0.0113/0.9)-1.209 × (0.993)age
